# Concentric and Eccentric Target MRI Signs in a Case of HIV-Associated Cerebral Toxoplasmosis

**DOI:** 10.1155/2018/9876514

**Published:** 2018-02-21

**Authors:** Adam D. Roche, Dominic Rowley, Francesca M. Brett, Seamus Looby

**Affiliations:** ^1^Department of Radiology, Division of Neuroradiology, Beaumont Hospital, Dublin 9, Ireland; ^2^The GUIDE Clinic (Genitourinary and Infectious Disease Department), St. James Hospital, Dublin 8, Ireland; ^3^Department of Neuropathology, Beaumont Hospital, Dublin 9, Ireland

## Abstract

Cerebral toxoplasmosis is one of the most common causes of focal brain lesions in immunocompromised patients, such as those with human immunodeficiency virus (HIV). Differentiating toxoplasmosis from other central nervous system (CNS) lesions provides a significant clinical challenge. Magnetic resonance (MR) imaging of the brain is key to prompt diagnosis and treatment of cerebral toxoplasmosis. Several specific signs on MRI of brain have been described in recent literature including the “concentric target sign” and “eccentric target sign.” We report a case of successfully treated HIV-associated cerebral toxoplasmosis in which both MRI signs were present simultaneously.

## 1. Introduction

Cerebral toxoplasmosis is an opportunistic infection caused by protozoan parasite* Toxoplasma gondii*, typically presenting in immunocompromised patients such as those with human immunodeficiency virus (HIV) [1]. Magnetic Resonance Imaging (MRI) plays an essential role in both the diagnosis and differentiation of this disease from other focal central neurological system (CNS) lesions that occur in HIV patients, such as primary CNS lymphoma, tuberculoma, and cryptococcosis infection [2]. Important MRI features described in cerebral toxoplasmosis include the “concentric target sign” in T2 weighted imaging and the “eccentric target sign” in postcontrast T1 weighted sequences [3, 4]. In this article, we present a case of a 31-year-old female with HIV-associated cerebral toxoplasmosis in which both of these of signs were present.

## 2. Case Report

This 31-year-old woman, with documented HIV infection for 8 years, presented with a four-day history of intermittent episodes of confusion associated with headache, fever, productive cough, and dyspnoea. Of note, the patient had declined treatment for HIV following her initial diagnosis and had not attended the outpatient department for several years.

On admission, she was moderately confused but had no focal neurological deficits or altered consciousness levels. Baseline laboratory tests revealed a very low CD4 cell count (14 cells/mm^3^ at 4%) and an HIV viral load of 206,548 copies/mL, indicating that the patient was significantly immunosuppressed and at risk for opportunistic infection. Other routine blood tests showed moderately raised inflammatory markers (CRP and ESR) with normal white cells and neutrophils. The patient's initial chest X-ray demonstrated patchy lower lobe infiltrates and she was commenced on treatment with trimethoprim-sulfamethoxazole for suspected pneumocystis pneumonia. Subsequent bronchoscopy with bronchoalveolar lavage (BAL) was negative for* pneumocystis jirovecii* and treatment for this infection was stopped with the patient receiving seven-day antibiotic treatment.

**Figure 1:**
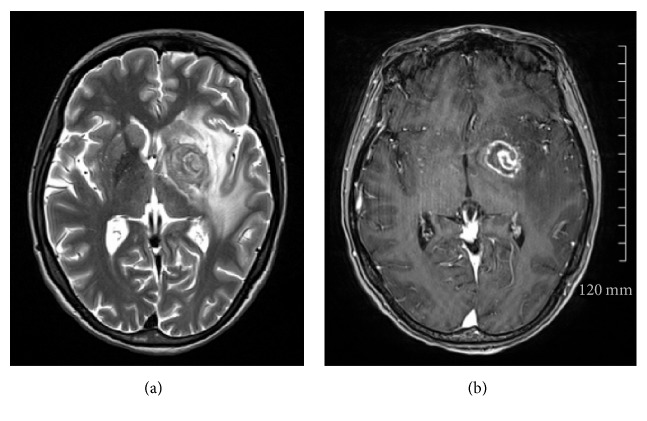
(a) T2 sequence demonstrating “concentric” target sign with concentric alternating hypointense and hyperintense rims. (b) Postcontrast sequence demonstrating “eccentric” target sign with a peripheral rim of enhancement and focal left lateral eccentric enhancing nodule.

**Figure 2:**
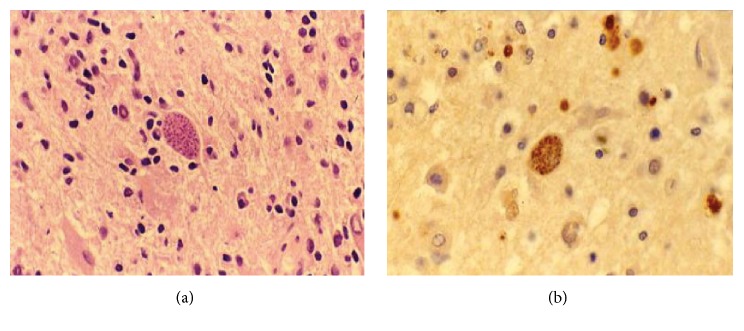
(a) Toxoplasma cyst demonstrated on H and E stain ×40. (b) Toxoplasma cyst confirmed on immunocytochemistry ×40.

Computerised tomography (CT) brain scan was performed on admission to investigate the cause of acute confusion and revealed a left basal ganglia lesion with mass effect. Differentials included toxoplasmosis and primary CNS lymphoma. MRI of brain confirmed a single left basal ganglia mass measuring 2.5 cm with alternating concentric T2 hypointense and hyperintense rims (Figure 1(a)) and postcontrast focal eccentric enhancing nodule that measured 1 cm (Figure 1(b)). These are considered both the “concentric” and “eccentric” target signs, which demonstrate specificity in the setting of cerebral toxoplasmosis. Biopsy of the lesion was performed and histopathological analysis confirmed toxoplasma cysts (Figure 2(a)) confirmed on immunohistochemistry (Figure 2(b)). There was no histological evidence of lymphoma. Further diagnostic testing such as serology and lumbar puncture was not required following detection of toxoplasmosis on biopsy.

The patient was commenced on treatment for toxoplasmosis, which involves a six-week course of antimicrobial therapy including sulfadiazine, pyrimethamine, and folinic acid. She responded well to treatment and her confusion resolved. Of note, while awaiting BAL results, the patient also completed a seven-day course of trimethoprim-sulfamethoxazole for suspected pneumocystis pneumonia, which is an alternative treatment choice for CNS toxoplasmosis. She was commenced on highly active antiretroviral therapy (HAART) and was counselled by the genitourinary service regarding the importance of compliance to her HIV treatment in the future.

CT brain scan was performed two days after biopsy and showed stable focal lesion with decreased vasogenic oedema. Repeat chest X-ray after two weeks showed complete resolution of infiltrate. She was eventually discharged from hospital four weeks after her initial presentation with outpatient follow-up arranged in the HIV clinic, at which point her CD4 count was noted to have risen to 129 cells/mm^3^ and 10% and her HIV viral load had reduced to 129 copies/mL.

## 3. Discussion

Despite significant advances in the prevention and treatment of HIV, the diagnosis, evaluation and subsequent management of CNS complications in patients with HIV remain a difficult clinical challenge. MR imaging of brain and spine is key in the investigation of immunosuppressed patients who present with neurological symptoms such as altered mental status [2]. The leading diagnostic considerations for CNS lesions with mass effect in a previously untreated HIV patient, as demonstrated in this clinical scenario, are cerebral toxoplasmosis and primary CNS lymphoma [5, 6]. Other CNS sequelae of HIV include cerebral tuberculosis, cerebral cryptococcus, and progressive multifocal leukoencephalopathy (PML).

Differentiating toxoplasmosis from CNS lymphoma is difficult, with significant therapeutic implications. Definitive diagnosis of toxoplasmosis requires a compatible clinical syndrome, detection of the organism in a biopsy specimen, and solitary or multiple intracerebral lesions with mass effect on MRI of brain [1]. Other diagnostic tests include serology for anti-toxoplasma IgG antibodies and CSF evaluation for evidence of* T*.* gondii*. Without treatment toxoplasmosis can be fatal and in the majority of cases, therapy is initiated after making a presumptive, rather than definitive, diagnosis of toxoplasmosis. Brain biopsy for ascertaining diagnosis is avoided if possible due to significant mortality risk; however because there is significant overlap between the imaging findings and clinical presentation, biopsy is often required [6].

Multiple rim-enhancing lesions in the basal ganglia favour a diagnosis of toxoplasmosis over CNS lymphoma and there are several key neuroimaging patterns seen in toxoplasmosis which guide differentiation from CNS lymphoma. On imaging, toxoplasmosis lesions are typically located at the corticomedullary junction and basal ganglia, while CNS lymphoma consists of solitary or few lesions with subependymal and subarachnoid spread [3–5]. One of the most commonly described findings of CNS toxoplasmosis is the postcontrast T1 “eccentric target sign” that has three alternating zones: an innermost eccentric enhancing core, an intermediate hypointense zone, and an outer peripheral hyperintense enhancing rim [3]. A more specific imaging pattern is the more recently described “concentric target sign” on T2 weighted MR imaging. This focal lesion has alternating concentric layers of T2 weighted hypo- and hyperintensities [4]. In a review of 14 cases of cerebral toxoplasmosis, the majority of patients (70%) had either one or both signs while a smaller number (36%) had both signs simultaneously [6]. Previous literature indicated that these two signs are rarely seen in the same lesion suggesting they reflect different pathological states of toxoplasma lesions in evolution.

The eccentric target sign correlates histologically with a central enhancing core produced by inflamed vessels at the sulci surrounded by concentric zones of necrosis producing peripheral enhancing ring [4]. The concentric alternating rings correspond pathologically to zones of haemorrhage and necrosis with foamy histiocytes and haemorrhage forming the outer zones [3].

Both the “concentric” and “eccentric” target signs were demonstrated in this case of confirmed cerebral toxoplasmosis which occurred in a significantly immunosuppressed HIV patient. The patient was commenced on antimicrobial therapy against* Toxoplasma gondii* and was treated successfully. This case demonstrates the importance of recognition of the key radiological features of CNS lesions in HIV patients to prevent delay of treatment.
